# Adaptor Protein Complexes in HIV-1 Pathogenesis: Mechanisms and Therapeutic Potential

**DOI:** 10.3390/v17050715

**Published:** 2025-05-16

**Authors:** Maria Elena Barone, Alexis Lim, Madison Woody, Parisa Taklifi, Fatema Yeasmin, Kequan Wang, Mary K. Lewinski, Rajendra Singh, Charlotte A. Stoneham, Xiaofei Jia, John Guatelli

**Affiliations:** 1 Department of Medicine, University of California San Diego, La Jolla, CA 92093, USA; eln.barone@gmail.com (M.E.B.); mlewinski@health.ucsd.edu (M.K.L.);; 2 VA San Diego Healthcare System, San Diego, CA 92161, USA; 3 Department of Biomedical Sciences, Florida State University College of Medicine, Tallahassee, FL 32306, USAxiaofei.jia@med.fsu.edu (X.J.); 4 Department of Chemistry and Biochemistry, University of Massachusetts, Dartmouth, MA 02747, USA

**Keywords:** adaptor protein complexes, HIV, Nef, Vpu, Gag, Env

## Abstract

Adaptor protein (AP) complexes are critical components of the cellular membrane transport machinery. They mediate cargo selection during endocytosis and intracellular vesicular trafficking. Five AP complexes have been characterized (AP1-5), and together their roles extend to diverse cellular processes including the homeostasis of membranous organelles, membrane protein turnover, and immune responses. Human Immunodeficiency Virus type 1 (HIV-1) and other lentiviruses co-opt these complexes to support immune evasion and the assembly of maximally infectious particles. HIV-1 Nef interacts with AP1 and AP2 to manipulate intracellular trafficking and downregulate immune-related proteins such as CD4 and MHC-I. Vpu also co-opts AP1 and AP2, modulating the innate defense protein BST2 (Tetherin) and facilitating the release of virions from infected cells. The envelope glycoprotein (Env) hijacks AP complexes to reduce its expression at the cell surface and potentially support incorporation into virus particles. Some data suggest that Gag co-opts AP3 to drive assembly at intracellular compartments. In principle, targeting the molecular interfaces between HIV-1 proteins and AP complexes is a promising therapeutic approach. Blocking these interactions should impair HIV-1’s ability to produce infectious particles and evade immune defenses, leading to novel antivirals and facilitating a cure.

## 1. Introduction to Adaptor Protein Complexes

Adaptor protein (AP) complexes are a five-member family of heterotetramers that play a fundamental role in intracellular trafficking [1,2]. These complexes support the selective transport of cargo between cellular compartments, ensuring the correct localization and function of membrane proteins. APs act as molecular bridges that connect cargo proteins to vesicle coat proteins (in at least two cases, AP1 and AP2, clathrin). They play a central role in vesicle formation by mediating the inclusion of specific transmembrane proteins destined for vectorial transport within the cell [2]. They are crucial for endocytosis and other post-Golgi vesicular transport mechanisms, including transport to lysosomes. Their ability to recognize specific sorting signals (short linear motifs—typically, but not limited to, YxxΦ- and ExxxΦ-sequences as well as acidic clusters) within the cytoplasmic domains of cargo proteins ensures the selectivity and fidelity of intracellular transport [3]. Disruptions in AP function are associated with neurodegenerative and hematologic disorders, as well as immune system dysfunction, as reviewed in [1,4].

APs interact with various regulatory proteins that modulate their activity, fine-tuning vesicle formation and cargo selection [1,5,6]. For example, phosphorylation of the medium (μ) subunit of AP2 causes the complex to adopt an “open” state, increasing its affinity for cargo binding [7,8,9]. Interactions with phosphatidylinositols on membrane surfaces recruit certain AP complexes to their specific sites of action, e.g., PIP2 recruits AP2 to the plasma membrane [10,11,12]. Understanding the interactions of APs with cargo proteins, other vesicle coat proteins, and regulatory molecules is essential for decoding the molecular mechanisms that underlie intracellular membrane trafficking and understanding how viruses including HIV-1 exploit them [13,14].

**Figure 1:**
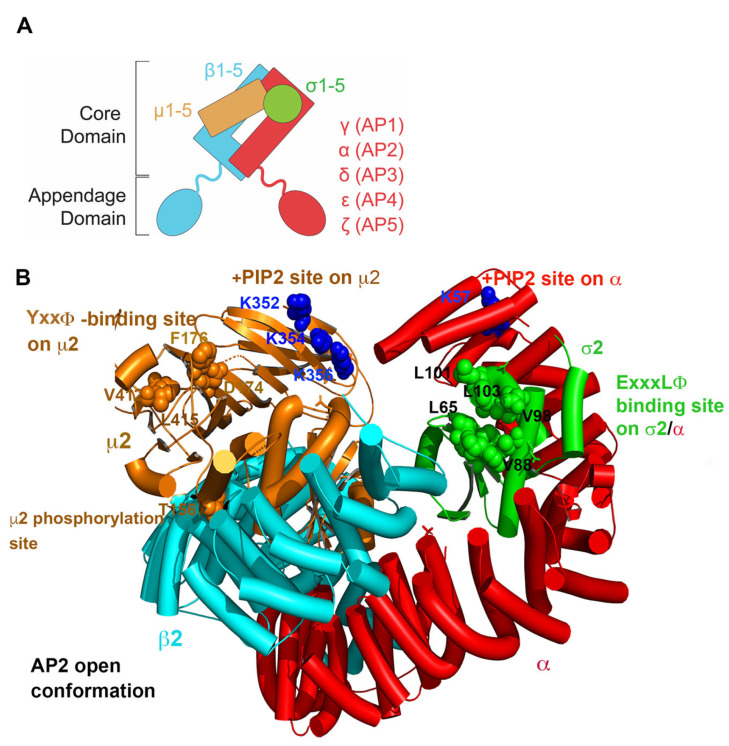
Subunit composition and example of an AP complex structure. (**A**) General diagram of the heterotetrameric AP complex. The core or trunk domain contains a large subunit that is specifically named for each complex (α, γ, δ, ε, or ζ), a large β subunit, a medium µ subunit, and a small σ subunit. The appendage domains interact with various regulatory proteins, and in the case of AP1 and AP2, with clathrin. (**B**) AP2 core domain shown in an “open” conformation in which the sites that bind “cargo proteins”—transmembrane proteins destined for specific vesicular transport—are accessible. The binding sites for the most common and best-understood sorting motifs within the cytoplasmic domains of cargo proteins are shown: YxxΦ sequences bind µ2, whereas ExxxLΦ sequences bind a site formed by α and σ2. In both sorting motifs, Φ is an amino acid with a bulky hydrophobic side chain, such as L, I, or V. Acidic cluster sorting motifs bind the µ subunits at basic patches (not shown here but see Section 2.3.3. for details). The phosphorylation site on µ2 (T156) is shown; phosphorylation leads to the “opening” of the complex. Also shown are the binding sites for the phospholipid PIP2, which facilitates the interaction of AP2 with the inner leaflet of the plasma membrane. Tubes indicate α-helices; ribbons indicate β-strands; spheres indicate binding sites. PDB code: 2XA7.

**Figure 2:**
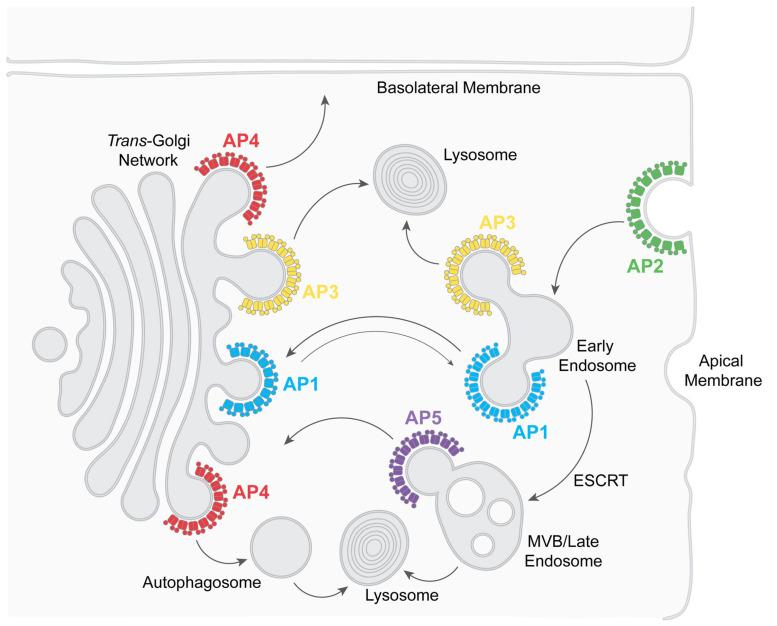
Physiologic roles of adaptor protein complexes in intracellular post-Golgi vesicular transport. See text for details. The double-arrow in the case of AP1 suggests bidirectional transport, but retrograde transport (back to the trans-Golgi network) predominates. “ESCRT” stands for Endosomal Sorting Complexes Required for Transport, a group of complexes and related proteins that move cargo proteins (canonically ubiquitinated but also non-ubiquitinated) to MVBs and eventual degradation. “MVB” is Multi-Vesicular Body, a precursor to lysosomes. “Lysosome” is lysosome or lysosome-related organelles, such as platelet granules or melanosomes.

APs form five complexes: AP1-5. These complexes share a similar general structure but have distinct subunit compositions and support specific functions [1,2] (Figure 1 and Figure 2). Each AP complex comprises four subunits: two large adaptins (β plus either α (AP2), γ (AP1), δ (AP3), ε (AP4), or ζ (AP5)), one medium-sized μ subunit, and one small σ subunit [1,2] (Figure 1).

Functionally, AP complexes support different aspects of vesicular transport (Figure 2). AP2 recruits transmembrane proteins into clathrin-coated pits at the plasma membrane, causing their endocytosis [7,15]. AP3 recruits transmembrane proteins in the trans-Golgi network (TGN) and early/recycling endosomes, sending them to lysosomes and lysosome-related organelles, such as platelet granules [4,16]. AP1, like AP2, interacts with clathrin but mediates transport between the TGN and the endosomal system, predominantly in a retrograde direction (bringing proteins back to the TGN) [15]. AP4 and AP5 are also implicated in specialized trafficking pathways: AP4 in transport to the basolateral plasma membrane of polarized cells, as well as to autophagosomes, and AP5 in the retrieval of proteins to the TGN and autophagosome flux [4,17,18,19]. Given their pivotal role in intracellular membrane trafficking, APs are, not surprisingly, hijacked by viruses [13,14].

This review highlights how HIV-1 manipulates AP complexes to facilitate virion assembly, infectivity, and escape from innate and adaptive host immunity. We specifically focus on how the viral proteins Nef, Vpu, Env, and Gag co-opt AP complexes to exploit or subvert host trafficking pathways, supporting replication fitness (Table 1). The activities of these HIV-1 proteins and their interactions with AP complexes are exemplified and elaborated at the immunologic, virologic, cellular, and structural levels. This information should foster insights into potential therapeutic interventions that disrupt viral replication and empower host immunity to clear the virus.

## 2. HIV-1 and AP Complexes

### 2.1. Modulating Innate and Adaptive Immunity

#### 2.1.1. Lentiviral Nef Interacts with AP1 to Modulate MHC-I, Rendering Infected Cells Less Susceptible to Virus-Specific Cytotoxic T Lymphocytes (CTL)

Nef is a small peripheral membrane protein that is associated with membranes through N-terminal myristoylation. Nef facilitates the formation of ternary super-complexes between host proteins and AP complexes, altering the trafficking pathways of host proteins in favor of the virus [13,20]. For instance, Nef hijacks the AP1 complex to prevent antigen-loaded MHC-I from reaching the cell surface, thereby reducing the susceptibility of infected cells to virus-specific cytotoxic T lymphocytes (CTLs) [21,22,23]. Instead of marking infected cells for destruction by CTLs, MHC-I is diverted by Nef from the TGN to lysosomes for degradation [23,24,25].

The exact mechanism by which the Nef/AP1/MHC-I interaction exerts effects at the cellular level remains uncertain, as the primary role of AP1 appears to be the retrieval of proteins to the TGN, rather than facilitating forward transport from the TGN to endosomes and lysosomes [26]. Additionally, AP3, the AP complex most closely linked to lysosomal targeting, is not known to participate in the degradation of MHC-I. Rather, a subpopulation of AP1 incorporating a specific isoform of the γ subunit, γ2 (AP1γ2), seems to mediate lysosomal targeting [27], along with contributions from COP-I [24], a vesicle coat complex that is similar to, but not classified as, a member of the AP family. Exactly how these interactions collaborate, and in what order, are unknown.

**Figure 3:**
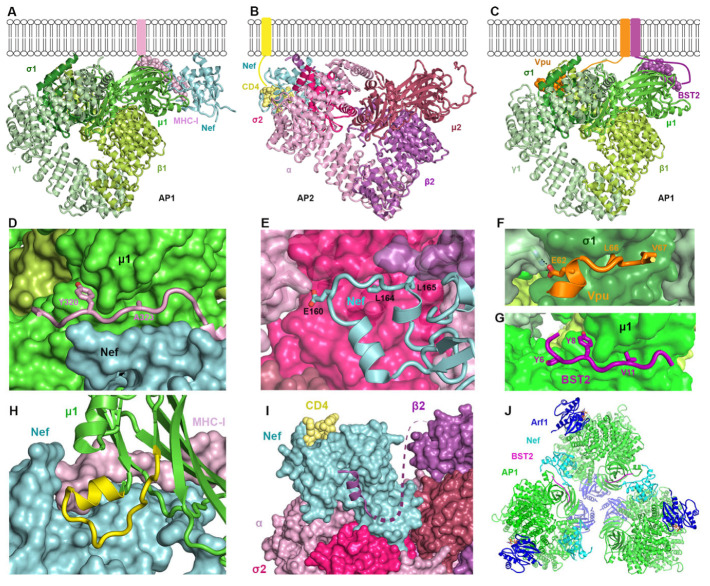
High-resolution structures provide biological insights into Nef- and Vpu-mediated hijacking of clathrin-associated AP complexes. (**A**) Structure of the MHC-I_CD_-Nef-AP1 complex, created by overlaying the Nef-MHC-I_CD_ -μ1_CTD_ structure (PDB:4EMZ) [28] with the hyper-unlocked AP1 from PDB: 4P6Z [53]. (**B**) Structure of the CD4_CD_-Nef-AP2 complex [41]. The μ2^CTD^, which was not included in PDB: 6URI, was appended through overlaying 6URI with the structure of open AP2 (PDB: 2XA7) [11]. (**C**) Structure of the BST2_CD_-Vpu_CD_-AP1 complex (PDB: 4P6Z) [53]. (**D**) MHC-I_CD_ binds at the Nef-μ1 interface. While MHC-I Y320 binds snugly into the binding site for tyrosine, the sidechain of A323 does not reach into the hydrophobic pocket of μ1. (**E**) Nef’s C-terminal loop binds extensively to σ2 and α subunits of AP2 partly through mimicry of the acidic dileucine sorting motif. (**F**) The acidic dileucine motif of Vpu_CD_ interacts canonically with the γ/σ1 subunits of AP1. (**G**) The YDYCRV sequence of BST2_CD_ binds into the tyrosine motif-binding pocket of μ1. (**H**) An otherwise-unstructured segment of μ1 (yellow) becomes ordered and adopts a helix-turn conformation when bound to Nef and MHC-I. (**I**) The N-terminal helix of β2, displaced from its original fold due to Nef-binding, is attracted to the Nef surface, which subsequently unlocks Nef and allows Nef’s N-terminal arm to swing over for binding CD4. (**J**) Trimer of the BST2_CD_-Nef-Arf1-AP1 complex (PDB: 6CRI) [90].

On the other hand, the structural biology of the Nef/AP1/MHC-I interaction is well understood (as detailed further below) [28]. The cytoplasmic domain of the MHC-I α chain contains the sequence YSQA, which interacts weakly at best with the YxxΦ-binding pocket on the µ subunit of AP1 (µ1). Nef leverages the YSQA sequence, compensating for the lack of a hydrophobic residue at the Y+3 position by bracketing the cytoplasmic domain of the MHC-I α chain between itself and µ1 (see Figure 3). This ternary interaction involves acidic residues in Nef but not the ExxxLL AP-binding motif that is required for the modulation of CD4 [29]. Overall, by recruiting MHC-I to AP1—acting as an “adaptor complex adaptor”—Nef misdirects MHC-I to the lysosome and prevents antigen presentation [13].

#### 2.1.2. Nef Interacts with AP2 to Modulate CD4, Rendering Infected Cells Less Susceptible to Antibody-Dependent Cellular Cytotoxicity (ADCC)

Nef co-opts the AP2 complex to remove CD4, the virus’s primary receptor, from the cell surface via endocytosis [30,31,32]. This action has several effects: it prevents cell death caused by the superinfection of already infected cells [33]; it increases viral infectivity by preventing CD4 from being incorporated into virions as they bud from the plasma membrane [34]; and it enables the evasion of immune surveillance at the level of ADCC (antibody-dependent cellular cytotoxicity) [35,36]. ADCC involves the recognition of the virus’s receptor-binding glycoprotein, Env, on the cell surface by specific antibodies. The Fc domains of these antibodies are then recognized by natural killer (NK) cells, which destroy the infected cells. The Nef-mediated downregulation of CD4 renders infected cells less susceptible to ADCC [35,36]. By preventing the interaction of CD4 with Env, Nef prevents newly made virions from being trapped on the cell surface by CD4, which would increase the amount of Env displayed there, and prevents conformational changes in Env induced by CD4 that expose epitopes (CD4-induced or CD4i epitopes), which are recognized by antibodies as being particularly effective in supporting ADCC [37]. Thus, similarly to MHC-I, by downregulating CD4, Nef protects infected cells from an immune response that would otherwise eliminate them. Following Nef-mediated endocytosis, CD4, like MHC-I, is sent to the lysosome for degradation. Similar cellular cofactors play roles in the lysosomal targeting of CD4, including the COP-I vesicle coat, AP1γ2, as well as ALIX, a scaffolding protein that facilitates interactions between the ESCRT (Endosomal Sorting Complexes Required for Transport) complexes [24,38,39]. ESCRT complexes mediate the transport of cargos that are canonically ubiquitinated but also non-ubiquitinated, and they drive the formation of multivesicular bodies (MVBs), which serve as lysosomal precursors) (Figure 3) [40].

As detailed below, Nef interacts with CD4 and AP2 very differently than with MHC-I and AP1. The interaction with AP2 is driven by an ExxxLL-binding motif in a flexible loop near Nef’s C-terminus. This sequence, like similar motifs in cellular transmembrane proteins, binds to a canonical site on AP2, formed by the σ2 and α subunits (Figure 1 and Figure 3). Unlike the case of the MHC-I α chain, the cytoplasmic domain of CD4 does not participate in binding to the AP complex. Instead, it binds to Nef. Nef acts as a connector, bridging CD4 and AP2 [41].

#### 2.1.3. Nef Interacts with AP1γ2 to Send MHC-I and CD4 to Lysosomes for Degradation

AP1 was initially described as the cofactor for the Nef-mediated diversion of MHC-I at the TGN (see Section 2.1.1), but a subset of AP1 complexes containing the γ2 subunit, AP1γ2, were subsequently shown to target MHC-I to the lysosome [27]. Thus, two AP1 variants participate in different steps of Nef-mediated MHC-I downregulation: the interaction of Nef with AP1γ1 recruits MHC-I at the TGN as the first step in preventing antigen-loaded MHC-I from being transported to the cell surface, and the interaction with AP1γ2 leads to its subsequent degradation in lysosomes [23,39]. Similarly, while Nef interacts with AP2 to stimulate the endocytosis of CD4 (see Section 2.1.2), CD4 is subsequently transported to the lysosome for degradation, in part via AP1γ2 [39].

#### 2.1.4. Vpu Interacts with AP1 and AP2 to Counteract the Interferon-Induced Protein BST2

The HIV-1 protein Vpu is a small type I transmembrane protein with nearly no luminal domain. It multimerizes with itself and cellular proteins through its transmembrane domain [42,43]. Its cytoplasmic domain interacts with cellular cofactors that facilitate the mis-trafficking and degradation of various cellular protein targets, including Bone Marrow Stromal Antigen-2 (BST2), also referred to as Tetherin [44,45]. BST2 is an interferon-induced protein found on numerous cellular membranes, including the plasma membrane [46]. It consists of two lipid anchors—a GPI anchor and a transmembrane domain—separated by an extended coiled-coil, allowing it to tether adjacent lipid bilayers together [47]. This enables BST2 to trap lipid-enveloped virions on the membranes from which they bud. In the case of HIV-1, BST2 traps nascent virions on the plasma membrane of infected cells, impeding their release and spread [44,45].

Vpu counteracts virion-entrapment by BST2 through a multifaceted mechanism: reducing BST2 expression at the plasma membrane, relocating BST2 away from budding virions within the plasma membrane, targeting BST2 for lysosomal degradation, and preventing the “forward trafficking” of newly synthesized BST2 to the cell surface, reviewed in [20]. Vpu binds BST2 through a transmembrane (TM)–TM interaction while engaging cellular cofactors, including AP complexes, with its cytoplasmic domain [43,48,49,50,51]. The efficient downregulation of BST2 from the plasma membrane requires AP2 [52]. Paradoxically, although interaction between Vpu and AP2 has been reported [49,53], Vpu does not increase the endocytic rate of BST2, indicating that it may act on BST2 that has already been endocytosed [52]. Vpu interacts directly with AP1 [53] (Figure 3). This interaction appears to have two consequences: it supports the ability of Vpu to inhibit the forward trafficking of BST2 from the TGN to the cell surface, and it displaces BST2 away from the viral structural proteins Gag and Env within the plasma membrane, preventing virion-entrapment [54]. At least two sequences in the cytoplasmic domain of Vpu are required for the displacement of BST2 away from forming virions: an ExxxLV motif in Vpu’s cytoplasmic domain [55], which binds the σ1 and γ subunits of AP1 [53], and a C-terminal sequence whose binding partner is unknown but whose function can alternatively be fulfilled by a clathrin-binding sequence [50,56]. Vpu also binds the µ subunit of AP1 through an acidic cluster motif (DpSGxxpS, where pS indicates phosphoserine) positioned just N-terminal of the ExxxLV sequence [51]. The endocytic activity of the Vpu cytoplasmic domain and the downregulation of BST2 from the cell surface by Vpu rely on both motifs and are inhibited by the depletion of clathrin and AP2 [49,51]. Notably, the phosphoserine acidic cluster of Vpu also binds the substrate adaptor β-TrCP, a subunit of the β-TrCP1/2-Skp1-Cullin1-F-Box (SCF) E3 ubiquitin ligase [48]. This interaction triggers the ubiquitination of BST2 and facilitates its eventual degradation in lysosomes [49,52,57,58]. The E3 ubiquitin ligase interaction also underpins Vpu’s ability to degrade CD4, although this occurs via an ERAD (ER associated degradation)-like mechanism that does not appear to involve AP complexes [59].

Virion-entrapment by BST2 has immunologic as well as virologic consequences. It increases the amount of Env on the cell surface and consequently sensitizes infected cells to ADCC [36,60]. It also induces the activation of NFĸB by aggregating a hemi-ITAM (immune-receptor tyrosine-based activation motif) within the BST2 cytoplasmic domain [61]. By alleviating virion-entrapment, Vpu counteracts these activities of BST2 through the mechanisms discussed above.

The structure of the Vpu-BST2-AP1 super-complex reveals yet another mode of cellular protein recruitment to an AP complex by an HIV protein [53]. Vpu and BST2 interact through their transmembrane domains, while each of their cytoplasmic domains binds to distinct sites on AP1: the ExxxLV motif of Vpu binds the σ1 and γ subunits, while the YDYCRV motif of BST2 binds the µ1 subunit [53] (Figure 3). Notably, the YDYCRV motif is absent in a short isoform of BST2 that results from translation from an internal initiator codon [62]. The absence of this motif renders short BST2 significantly less sensitive to antagonism by Vpu, potentially due to the lack of this AP-binding sequence.

### 2.2. Assembly and Release of Infectious Virions

#### 2.2.1. Nef-Mediated Modulation of CD4 and SERINC Proteins via AP2 Increases Virion Infectivity

As reviewed above, Nef removes CD4 from the cell surface by triggering its endocytosis, linking the cytoplasmic domain of CD4 to the AP2 complex. If not downregulated by Nef (and Vpu), CD4 inhibits HIV-1 infectivity by incorporating into virions and binding to Env [34,63]. Similarly to CD4, the host cell proteins SERINC5 and SERINC3 also inhibit HIV-1 infectivity; SERINC5 plays the more significant role [64,65]. The mechanism through which SERINC proteins inhibit infectivity is not well understood. However, like CD4, they incorporate into virions and interfere with the Env-mediated fusion of virions with target cells [66,67]. SERINC proteins are removed from the plasma membrane by Nef via AP2, followed by lysosomal degradation [68]. SERINC3 and SERINC5 are multi-pass transmembrane proteins, each containing an acidic cluster in their longest cytoplasmic loop that binds to AP complex µ subunits in vitro [69,70]. Paradoxically, these potential AP-binding motifs are dispensable for antagonism by Nef. In the case of SERINC5, the acidic cluster instead appears to confer resistance to Nef [70]. The structural basis of the putative Nef/SERINC/AP2 interaction remains unknown.

#### 2.2.2. While HIV-1 Vpu Uses AP1 and AP2 to Counteract Virion Entrapment by BST2, SIV Accomplishes This Using Nef and AP2

As reviewed above, Vpu co-opts, among other cellular cofactors, AP1 and AP2 to counteract BST2 and stimulate the release of virions from cells. In most Simian Immunodeficiency Virus (SIV) strains, which lack Vpu, BST2 antagonism is provided by Nef and occurs via AP2. The interaction between SIV Nef and AP2 depends on the Nef ExxxLΦ AP binding motif [71]. Somewhat reminiscent of the effects of HIV-1 Nef on AP2 when bound to CD4 (discussed below), SIV-Nef refolds the N-terminus of the β2 subunit, creating a binding pocket for a sequence in the cytoplasmic domain of simian BST2 [72].

#### 2.2.3. Env Interacts with AP Complexes: Immune Evasion and Virion-Incorporation

HIV-1 Env, the virus’s receptor-binding glycoprotein, contains sequences within its cytoplasmic domain that bind AP complexes, most notably a YxxΦ sequence that binds µ subunits and contributes to immune evasion and virion incorporation [73,74]. Env is a type I transmembrane protein that is translated in the ER and glycosylated there and in the Golgi, where the gp160 precursor protein is cleaved into the gp41 (transmembrane and cytoplasmic domains) and gp120 (surface) subunits by Furin-like proteases [75] before transiting to the plasma membrane. The HIV Env gp41 cytoplasmic domains contain a YxxΦ tyrosine-based motif (YSPL), which mediates endocytosis via clathrin-coated pits [73,76,77,78]. This motif interacts with μ2 (AP2), as well as μ1 (AP1) and μ3A (AP3) [74]. We reported that an intact YSPL motif is required for optimal infectivity and virion incorporation of Env, suggesting that AP complexes may play a role in targeting Env to the forming virion [79]. A dileucine motif in the cytoplasmic domain also contributes to virion incorporation [80]. This motif reportedly binds AP1, affecting the intracellular localization of Env without altering its endocytic rate [81]. How these AP interactions support the incorporation of Env into virions, which occurs during budding from the plasma membrane, remains unclear. One hypothesis suggests that endocytosis and recycling are necessary to properly target Env to viral assembly sites [82]. The presence of endocytic signals in Env might seem paradoxical, since the plasma membrane is where HIV assembly and budding occur. However, Env is the only viral protein expressed at the cell surface, and it is the sole target for ADCC. By reducing the display of Env at the cell surface, the virus likely minimizes Env-epitope exposure while allowing for virion incorporation. The AP-binding sequences in Env presumably strike this balance. Remarkably, a deletion including the tyrosine of the YSPL-analogous sequence in SIV (YRPV) yields a mutant that initially replicates well in experimentally infected macaques but is ultimately more effectively controlled and less pathogenic, supporting the importance of AP-mediated trafficking of Env [83]. Potentially, the kinetics of replication and pathogenesis in this model favor an immune-evasion role rather than a direct virologic role for the AP interaction. In HIV-2, which like most SIVs lacks Vpu, BST2 antagonism is provided by Env and requires the tyrosine of the YRPV sequence; consistent with this, the stimulation of virion release by HIV-2 Env requires AP2 [84,85].

#### 2.2.4. Gag Interaction with AP3: Support of Virion Assembly at MVBs

AP3 has been reported to play a role in HIV-1 Gag trafficking and virion assembly in multi-vesicular bodies (MVBs), a putative site of viral assembly that is an alternative to the plasma membrane and has been posited in myeloid cells such as macrophages [86]. Gag is a multi-domain precursor protein of HIV-1 that includes matrix (MA), capsid (CA), nucleocapsid (NC), and p6. The evidence for a direct interaction between AP3 and Gag is conflicting: initially reported as an interaction between the δ subunit of AP3 and the MA of Gag using a yeast-two-hybrid assay [86], evidence for direct binding was not obtained by subsequent studies using recombinant proteins and NMR [87]. Nevertheless, consistent with a functional requirement for AP3, Hermansky–Pudlak syndrome type 2 cells, which encode a defective AP3B1gene, support HIV-1 virion release inefficiently [88]. In addition to AP3, AP5 has been reported to support the release of HIV-2 virions [89].

### 2.3. Structural Basis of HIV-1 AP Interactions

#### 2.3.1. How Nef and Vpu Involve Sequences Both in Their Cellular Targets and in Themselves to Interact with AP Complexes

We established high-resolution structures that reveal the intricate details of how Nef and Vpu hijack the clathrin adaptor protein complexes AP1 and AP2 (Figure 3A–C). A common theme in these interactions is that the sites on the AP complexes that are typically used to bind the linear sorting motifs of cellular proteins are co-opted by the viral proteins. For the Nef-mediated downregulation of MHC-I, the site on the μ1 subunit of AP1 that binds tyrosine motifs of sequence YxxΦ is exploited [28]. Y320 of MHC-I binds the pocket “designed” for the tyrosine of YxxΦ motifs (Figure 3D). However, the Φ-binding pocket on μ1 is not occupied because the MHC-I residue facing this pocket, A323, does not supply the required bulky hydrophobic sidechain (Figure 3D). Nef “rescues” this less-than-ideal binding between MHC-I and μ1 by making direct contact with both proteins at their interface and coordinating a three-way cooperative binding (Figure 3D). In contrast, to recruit CD4 into endocytic vesicles, Nef utilizes the binding site for the acidic dileucine (ExxxLL) sorting motifs found on AP2 and formed by the σ2 and α subunits [41]. Here, Nef directly mimics the acidic dileucine sorting motifs found in cellular proteins: the ExxxLL sequence within the C terminal loop of Nef binds into the sorting motif-binding site. The rest of Nef’s C-terminal loop further engages with the σ2 subunit (Figure 3E). This tight association enables a series of conformational changes—both in Nef and in the β2 subunit of AP2—, which subsequently lead to the recruitment of CD4 into a pocket on the opposite side of Nef (Figure 3E). In the case of BST2 modulation by Vpu, both sorting motif-binding sites on AP1 are involved [53]. While Vpu and BST2 interact with each other through their transmembrane helices, their cytoplasmic domains bind to separate locations/subunits of AP1: Vpu interacts with the γ and σ1 subunits by mimicking an acidic dileucine-sorting motif via its ExxxLV sequence (Figure 3F), while BST2 binds to the tyrosine motif-binding site on the μ1 subunit of AP1 via its YDYCRV sequence (Figure 3G). Thus, by taking advantage of the natural binding sites on AP complexes and adding additional interactions to them, some of which induce conformation changes, Nef and Vpu repurpose APs to target and mistraffick cellular proteins.

#### 2.3.2. How Nef, Vpu, and Their Targets Change the Conformation of AP Complexes

The membrane- and cargo-binding activity of AP complexes is regulated by conformational changes induced by regulatory molecules, including the GTPase Arf1 and phosphoinositides [12,91]. Vpu and Nef also induce conformational changes in clathrin adaptor proteins, in some cases “opening” the complex to facilitate their interaction with it. For example, the short section of the μ1 subunit of AP1 from residue 215 to 233 is flexible and unstructured when not bound to cargo [92]. However, in the complex formed between Nef, μ1, and the MHC-I α chain cytoplasmic domain, this region of μ1 becomes well-ordered, and part of it forms the pocket that binds and recruits MHC-I [28] (Figure 3H). In downregulating CD4, Nef induces a dramatic conformational change in the β2 subunit of AP2 [41] (Figure 3I). Upon association with AP2 through interactions with its C-terminal loop (Figure 3E), Nef invades the space normally occupied by the N-terminal portion of β2. The N-terminal part of β2 becomes partially unfolded: in the high-resolution structure the four helices expected at the N-terminal end of β2 are not observed [41]. Instead, the most N-terminal helix relocates and binds to a Nef surface (Figure 3I). This helix^β2^–Nef binding frees Nef’s N-terminal flexible loop, allowing it to adopt a new conformation and directly bind with CD4. These structural findings illustrate how Nef utilizes relatively flexible parts of the AP complex, remodeling them into new conformations to suit its needs.

Nef also changes the oligomeric state of AP1. Nef, when N-terminally fused to either the cytoplasmic domain of BST2 or the MHC-I α, induces the formation of AP1 trimers in the presence of the GTPase Arf1 (Figure 3J) [90,93]. These AP1 trimers further associate into hexamers, the dimension of which matches the hexagons of clathrin coats. This suggests that Nef promotes the assembly of clathrin coats [93]. Subsequent data showed that AP1, Arf1, Nef, and MHC-I form coats on tubulated membranes in the absence of clathrin [94]. Although Nef is not required for the formation of these coats, it localizes to them, which likely enables the efficient recruitment of MHC-I. The lattice of the tubular coat is incompatible with clathrin-binding, but it could transition into a clathrin-like geometry, leading to the formation of a clathrin coat [94]. These data, using recombinant proteins, are potentially consistent with the Nef’s ability to stimulate the formation of clathrin-coated pits and to stabilize the attachment of AP complexes to membranes in living cells [95,96].

Vpu and BST2 together induce a highly open conformation of AP1 that exposes the binding sites for both the BST2 YDYCRV µ1-binding motif and the Vpu ExxxLV σ1/γ-binding motif [53]. This conformation is even more “open” than that induced by the GTPase Arf1 alone [53]. The same conformation—namely, the hyper-unlocked state—was subsequently observed in the AP1 trimers induced by BST2_CD_-Nef [93] (Figure 3J). These results suggest that both Vpu and Nef can stabilize a hyper-unlocked state of AP1, presumably for the more efficient recruitment of their targets into clathrin coats.

#### 2.3.3. How Nef and Vpu Informed on the Binding Partner of Acidic Cluster Sorting Motifs

Acidic cluster sorting motifs were first described in the cytoplasmic domain of the cellular endoprotease Furin [97], which localizes at steady-state to the TGN. Initially, the mode of interaction of acidic clusters with AP complexes was proposed as indirect, mediated by a cytosolic adaptor, PACS-1 [98]. The Nef/MHC-I α chain/µ1 complex structure instead revealed a direct interaction between an acidic region on Nef and a basic region on µ1 [28]. This raised the possibility that the interaction of acidic clusters with AP complexes in general was direct and mediated by µ subunits. The acidic cluster in Nef is primarily composed of glutamic acid residues, whereas that of Furin is made of glutamic and aspartic acid residues, as well as phosphorylated serines, a similar composition to that of the phosphoserine acidic cluster in Vpu. Ultimately, we found that the cytoplasmic domains of Furin, Vpu, SERINC3, and SERINC5 all directly bound µ subunits via their phosphorylated acidic clusters [51,69,70]. The conclusion that acidic clusters utilize the µ subunits of AP complexes was further supported in the case of Furin thorough a genetic screening [99]. Notably, µ subunits contain several basic patches, but other than the case of Nef, no structural data show exactly how acidic clusters interact with them.

## 3. Controversies, Open Questions, and Future Directions

Many important questions remain to be answered regarding the interactions between adaptor protein complexes and HIV-1 membrane-associated proteins. These concern both the structural basis of the interactions and their functional consequences and importance. For example, notwithstanding the substantial data supporting an interaction between Nef and AP3 [96], no AP3-dependent Nef-activities have been identified. Similarly, we are not aware of any data for or against a role for AP4 in the activities of HIV-1 proteins. On the other hand, while the Nef-AP2 interaction underlies several well-described activities, including the removal of both CD4 and SERINC5 from the cell surface (among others), a yet-to-be-identified cellular protein appears to be modulated by Nef Via AP2 to enhance the replication rate of HIV-1 [20,100].

The relationship between Vpu and AP complexes is only partially understood. While AP2 plays a cofactor role in the activity of Vpu as an antagonist of BST2, whether Vpu directly interacts with AP2 in cells is unclear. The structure of the Vpu-BST2-AP1 complex has been partially solved (Figure 3), but most of the structure of Vpu in the complex remains undefined, including how the acidic cluster (DpSGxxpS) binds µ1. Functionally, AP1’s role as a cofactor in the Vpu-mediated inhibition of the forward trafficking of BST2 is consistent with its primary trafficking pathway: the retrieval of cargoes to the TGN. On the other hand, its support of the Vpu-mediated displacement of BST2 from viral assembly sites within the plasma membrane suggests an atypical intracellular location for AP1-activity [54]. Vpu’s apparent ability to interact with AP complexes in two different modes—one using its ExxxLV sequence to bind σ1/γ and the other using its acidic cluster to bind µ1—is of uncertain significance [51]. Whether these two modes of interaction relate to the modulation of different host cell proteins by Vpu, similarly to how Nef uses distinct modes of interaction with AP complexes to modulate CD4 and MHC-I, is unknown.

The relationship between Gag and AP3 (as well as AP1 and AP2) seems open to exploration. Functional data indicate a role for AP3 in virion assembly, but direct binding between Gag and AP3 has not been observed [87]. Moreover, the underlying model of HIV-1 assembly in multivesicular bodies, to which AP3 could reasonably support the transport of Gag, is currently disfavored, even in myeloid cells where virions accumulate in intracellular virus-containing compartments [101,102]. In addition to AP3, both AP1 and AP2 reportedly interact with Gag, and knockdown data suggest that AP1 and AP3 might support overlapping aspects of viral assembly and release [103,104]. The exact mechanisms by which these AP complexes support Gag trafficking and/or viral morphogenesis remain to be defined.

The interaction of HIV-1 Env with AP complexes seems straightforward insofar as the YxxΦ-mediated interaction with µ2 supports the endocytosis of Env, allowing for the evasion of ADCC. Still, the functions that attend the interactions of Env with AP1 and AP3 are less clear. Moreover, exactly how these AP interactions facilitate the incorporation of Env into virions—if they do—remains to be fully elucidated.

Finally, the structural models of the above interactions derive from recombinant protein complexes; understanding their forms in cellulo is a cutting-edge question.

## 4. Opportunity for Therapeutic Intervention

Therapeutic intervention in HIV-1 AP interactions could yield direct antiviral activity through inhibiting virion assembly and infectivity, or it could inhibit the virus’s immune evasion activities, facilitating the clearance of infected cells via CTL activity or ADCC. Approaches include targeting specific structural features of these interactions, screening for potential inhibitors using interaction or functional assays, and using peptide mimics to decoy viral proteins from their cellular targets or the AP complexes themselves.

An intriguing structural target is within the complex formed by Nef, μ1, and MHC-I, in which the cytoplasmic domain of the MHC-I α chains fits into a narrow groove formed by Nef and μ1 (Figure 3) [28]. In principle, a small molecule that supports and fills this groove could compete with MHC-I, inhibiting modulation by Nef. An apparent inhibitor of this interaction is concanamycin A, which was identified in a functional screening and decreased formation of the Nef-MHC-1-AP1 complex in living cells; however, it did not block formation of the complex using recombinant proteins, rendering its mechanism of action unclear [105].

In addition to directly targeting these protein–protein interactions, interference with the activities and regulation of AP complexes at the cellular level should render them unable to act as viral cofactors. This approach would likely have deleterious effects on cellular homeostasis and function, rendering it unsuitable for the continuous and indefinite application that typifies current antiretroviral therapy. Nonetheless, such approaches could be part of a short-term cure strategy that aims to sensitize infected cells to elimination by interfering with the AP-dependent immune evasion. To this end, regulatory enzymes are potential targets. For example, AP1 and AP3 require the activity of the GTPase Arf1 to cycle on and off membranes [106,107]. The adaptor-related kinases GAK (auxillin-2) and AAK1 are also attractive and should be tractable to inhibition by small molecules. GAK is required for the uncoating of clathrin-coated vesicles [108,109]. The inhibition of GAK might indirectly interfere with the activities of Nef and Vpu. AAK-1 (adaptor associated kinase 1) phosphorylates the µ subunit of AP2, inducing the “open” conformation associated with cargo-binding [7,110]. An AAK-1 inhibitor could, in principle, interfere with the Nef-mediated endocytosis of CD4 and the intrinsic endocytosis of Env, increasing the display of Env and CD4-induced epitopes at the cell surface with consequent sensitization to ADCC. Notably, an AAK1 inhibitor reached phase II clinical trials as a treatment for neuropathic pain, suggesting its potential repurposing in HIV cure strategies [111,112].

## 5. Conclusions

Adaptor proteins (APs) are essential for the intracellular trafficking of membrane proteins. Their interactions with HIV-1 proteins provide critical insights into how the virus evades host immunity and efficiently produces progeny virions of optimal infectivity. The HIV-1 proteins Nef, Vpu, Env, and Gag hijack AP complexes, either for their transport or to misdirect cellular proteins within the endosomal system, ultimately enhancing viral fitness and pathogenesis. Although significant progress has been made in understanding these interactions, many questions remain regarding the structural basis of AP–HIV-1 interactions and the role of different AP complexes in viral replication. Future research should address these uncertainties and investigate whether targeting AP-dependent pathways can yield novel antiviral strategies, with special attention to their potential role in a cure. Regardless of whether such therapeutic possibilities come to fruition, the study of adaptor protein complexes and HIV-1 exemplifies how immunology, virology, cell biology, and structural biology continue to inform one another.

## Figures and Tables

**Table 1:** Key Interactions of AP complexes with HIV-1 proteins and their functional consequences.

Adaptor Protein Complex	Cellular Functions	Clathrin-Association	Interactions with HIV-1 Proteins	HIV Functions Supported or Affected
AP1 (Adaptor protein complex 1)	Transport between endosomes and the trans-Golgi network (TGN), mostly retrograde	Yes	Nef, Vpu, Env, Gag	Modulation of class I MHC Modulation of BST2 (Tetherin) Env trafficking, assembly
AP2 (Adaptor protein complex 2)	Endocytosis	Yes	Nef, Vpu, Env, Gag	Downregulation of CD4 Downregulation of BST2 Endocytosis of Env, assembly
AP3 (Adaptor protein complex 3)	Transport to lysosomes and lysosome-related organelles	Controversial	Nef, Gag	Virion assembly in MVBs/intracellular virus containing compartments
AP4 (Adaptor protein complex 4)	Transport to the basolateral surface of polarized cells. Transport to pre-autophagosomes	No	Unknown	Unknown
AP5 (Adaptor protein complex 5)	Retrieval to the TGN; transport of proteins involved in autophagic flux	No	Unknown	Virion release (HIV-2)
